# Floating of the lobes of mosquito (*Aedes togoi*) larva for respiration

**DOI:** 10.1038/srep43050

**Published:** 2017-02-20

**Authors:** Seung Chul Lee, Jun Ho Kim, Sang Joon Lee

**Affiliations:** 1 Department of Mechanical Engineering, Pohang University of Science and Technology, Pohang Gyeongbuk, Republic of Korea; 2 Center for Biofluid and Biomimic Research, Pohang University of Science and Technology, Pohang Gyeongbuk, Republic of Korea

## Abstract

Mosquito (*Aedes togoi*) larva has to float its siphon on the water surface to breathe air. To elucidate the floating mechanism of the siphon, morphological structures, especially the flap-like lobes and spiracle of the siphon, were observed. Wettability and dynamic behavior of the lobes on the water surface were also experimentally examined. The lobes formed a hollow cone shape under water and expanded on the water surface. The spiracle was located at the base of the cone. The lobes exhibited hydrophobic wettability. During floating process, the lobes were spread into a triangular shape in apical view, and the spiracle was exposed to air. When they were submerged in water, the lobes were folded into a cone shape to seal the spiracle and protect it from water penetration. These dynamic processes occurred at the water surface. In this study, the floating mechanism of the lobes for respiration was described based on the cross-sectional morphology of the lobes with the pressure induced by surface tension and the hydrostatic pressure proportional to depth. This study would be helpful in elucidating the behavior of mosquito larvae near the water surface and may facilitate the formulation of methods to control mosquito larvae.

Mosquitoes have been studied intensively, because some of them are important vectors spreading crucial diseases to humans, such as malaria, dengue, chikungunya and Zika virus. Despite of intensive research, mosquito-borne diseases still seem threat the global public health. Thus the control of mosquitoes to prevent the transmission of diseases is a major issue. For mosquito control, the larva stage might be an attractive target, because mosquito larvae are restricted in water where they colonize, while adult mosquitoes can evade the control through the behavioral strategies^1^,^2^. The control methods for mosquito larvae include biological control using predators or parasites^3^, chemical control using insecticide^1^, environmental managements by eliminating the breeding sites^4^ and physical control using mechanical means^4^,^5^. Mosquitoes have been considered to unlikely develop the resistance to the physical control means^4^. To suppress mosquito larvae physically, oil, surface film and polystyrene beads floating on the water surface have been applied to the breeding sites^4^. These physical control methods mechanically disturb the contact of larvae with air above the water surface to prevent the effective respiration^4^. The detailed understanding on the respiration mechanism of mosquito larvae would be helpful for the design of these control works.

Oxygen cannot be easily obtained in water without special strategies. Aquatic insects have developed breathing strategies depending on their structures and wettability^6^,^7^. They can obtain oxygen directly from air by making contact with the water surface^7^,^8^, or obtain oxygen dissolved in water through tracheal gills^7^,^9^ or through permanent thin air layer on exterior^6^. Insects can also remain underwater for a finite period by consuming temporary air bubble whose volume decreases over time^10^. The *Aedes* mosquito larva obtains air through an air tube called a siphon. The larva floats the apical part of its siphon during breathing^8^,^11^. The apical part of the siphon has five flap-like lobes and an air hole called a spiracle^8^,^11^. The larva cannot float on the water surface when the lobes are removed^8^,^11^,^12^. Brocher^13^ reported the importance of the wettability and surface tension for floatation of *Culex* mosquito larvae with flap-like lobes. Therefore, the lobes presumably play a functional role in floating on the water surface. However, relatively little information is available on how the lobes float on water and contact air, the dynamic behavior of these lobes at the water surface, and the interaction between the lobes and water under *in vivo* conditions.

In this study, the floating of a mosquito (*Aedes togoi*) larva by its lobes on the water surface was experimentally investigated. The morphological structures of the lobes and spiracle were observed by a scanning electron microscope (SEM) and synchrotron X-ray micro-computed tomography (CT). The wettability of the lobes was estimated by observing the shape of the air bubble on them. Furthermore, the dynamic behavior of the lobes on the water surface was recorded using a high-speed camera. The model for the floating lobes was described based on these systematic observations. This study would be helpful for understanding the behavior of mosquito larvae on the water surface.

## Results

### Morphological features of lobes of the siphon

**Figure 1:**
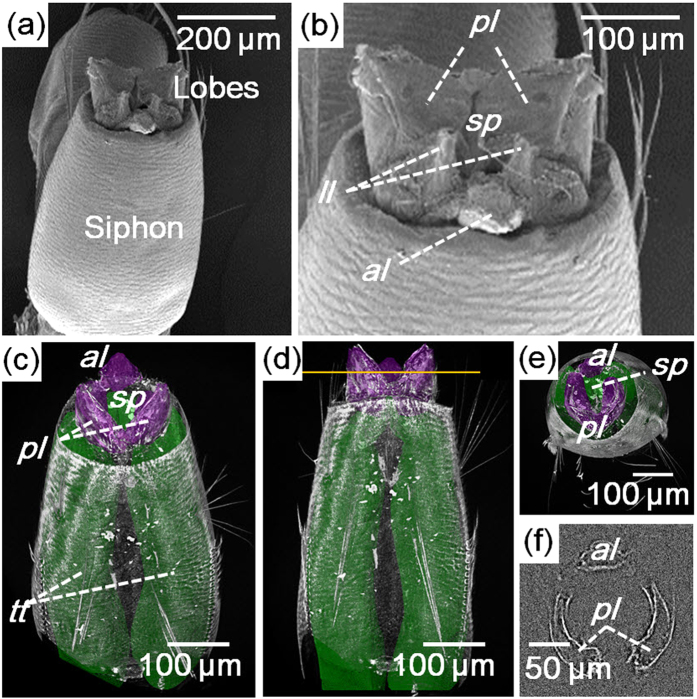
Morphology of the apical part of the siphon of *Aedes togoi* fourth instar larva. (**a,b**) SEM images of the siphon. (**a**) Outline of the siphon and lobes. (**b**) Apical part of the siphon. Spiracle is surrounded by five lobes (two pairs of lateral and posterior perispiracular lobes and a single anterior perispiracular lobe). (**c**) The 3D reconstructed tomographic image of the siphon showing tracheal trunk (green) and three lobes (purple). (**d**) Lateral and (**e**) top views of the siphon. (**f**) Cross-sectional view of the three lobes cut along the yellow line. *al*, anterior lobe; *ll*, lateral lobe; *pl*, posterior lobe; *sp*, spiracle; *tt*, tracheal trunk.

The structures of the siphon, especially the apical part, of the *Aedes togoi* larva were observed using an SEM (Fig. 1a,b). The body of the siphon is a blunt cylindrical tube tapering to the end with a circular edge^8^ (Fig. 1a,c). Figure 1b shows the apical part of the siphon with partially expanded lobes. Five lobes were placed inside the circular edge (Fig. 1b). A pair of posterior lobes showed the largest size. An unpaired anterior lobe was placed on the other side. Another pair of lateral lobes displayed the smallest size and placed between the anterior and posterior lobes. The spiracle was surrounded by these five lobes^8^,^11^.

The three-dimensional structure of the siphon with partially spread lobes was analyzed using synchrotron X-ray micro-CT (Fig. 1c–f). The anterior and posterior lobes were clearly observed in the captured CT images, but the boundaries of lateral lobes were ambiguous. Therefore, the anterior and posterior lobes were mainly investigated in this study. The shape of three main lobes (purple-colored structures in Fig. 1c–e) resembled the petals of a flower. The height of the lobes was approximately 112 μm. Figure 1f shows the cross-sectional view of the three main lobes cut at the line in Fig. 1d. The cross section of each lobe was arc shaped. These arc-shaped lobes surrounded the spiracle connected to the tracheal trunk inside the siphon tube (Fig. 1e).

**Figure 2:**
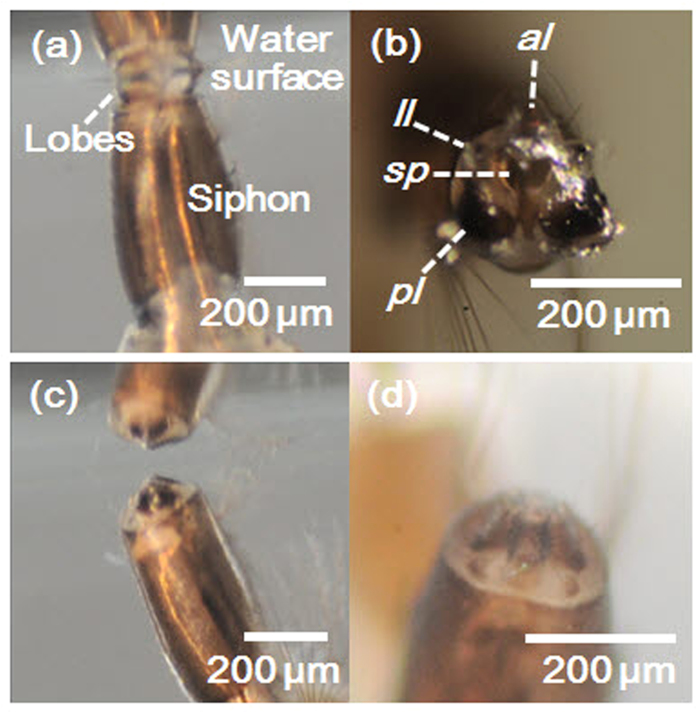
Magnified images of the siphon of the mosquito larva under water. (**a,b**) Siphon of a floating larva. (**a**) Lateral view of the siphon. Expanded lobes hang from the water surface. (**b**) The apical part of the floating siphon showing expanded lobes and open spiracle. (**c,d**) Siphon of a submerged larva. (**c**) Lobes are folded into a conical shape. (**d**) Spiracle is sealed by the lobes.

In addition, the morphology of the siphon of the living larva was observed when they floated on the water surface (Fig. 2a,b) and submerged in water (Fig. 2c,d). They floated on the water by touching the water surface with the lobes (Fig. 2a). In this floating state, the lobes widened the gaps between themselves and expanded to a triangular shape in apical view (Fig. 2b). Figure 2b shows that the spiracle of the floating siphon was open. When the larva was submerged, the lobes contracted and folded into a cone-like shape (Fig. 2c). Thus, the gaps between lobes disappeared, and the spiracle was sealed by the lobes (Fig. 2d).

### Wettability of the apical part of the siphon

**Figure 3:**
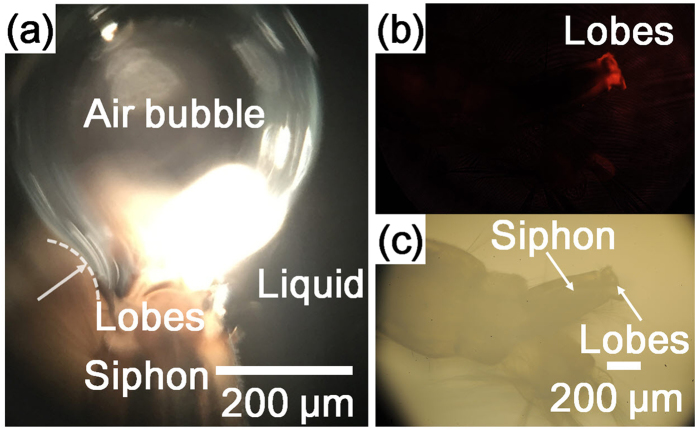
Wettability of the siphon. (**a**) Air bubble released from the spiracle forms a negative curvature (white dotted line) on the lobes. (**b**) Fluorescent image of the larva stained with Nile red, a hydrophobic fluorescence probe. (**c**) Optical image of the same larva observed using a light microscope.

The interface between air and liquid on the apical part of the siphon was investigated to determine the wettability of the apical part of the siphon. The shape of the air bubble on the submerged lobes exhibited a negative curvature (white dotted line in Fig. 3a). This result can indicate that the surface had hydrophobic wettability. The air bubble can have negative curvature when the contact angle of water on the substrate is greater than 90°.

The wettability of the apical part of the siphon was also investigated using Nile red, a fluorescent hydrophobic probe^14^,^15^. The fluorescence image of the larva stained with Nile red shows a fluorescent apical part of the siphon, indicating that the apical of the siphon is hydrophobic (Fig. 3b). An unstained larva was also observed to check the effect of autofluorescence, which was found to be negligible because the fluorescence image of the unstained larva was dark.

### High-speed imaging of the dynamic behavior of lobes

**Figure 4:**
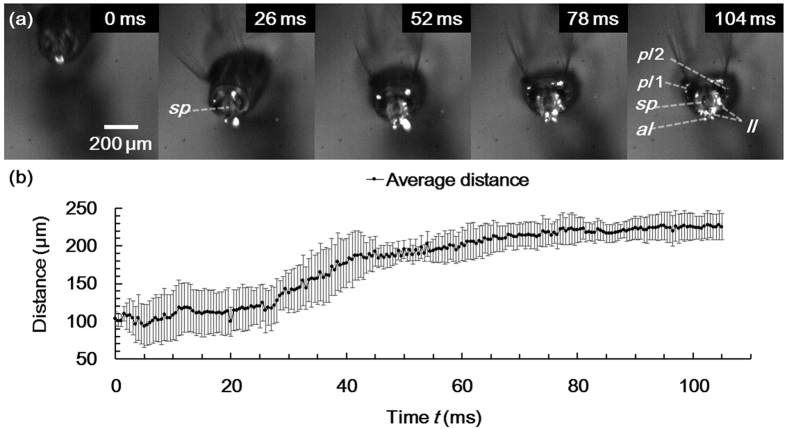
Floating process of the siphon observed by a high-speed camera. This process takes approximately 105 ms. The start (*t* = 0 ms) and end (*t* = 105 ms) points are defined as the instants at which the lobes begin to touch water surface and are fully spread, respectively. (**a**) High-speed images extracted at intervals of approximately 1/4 *T* (26 ms). (**b**) Temporal variation of average distance (mean ± s. d.) between the ends of the three lobes (*pl*1*, pl*2*, al*) during the floating process.

The dynamic behavior of the lobes was observed during floating process (defined in Methods). The lobes touched the water surface with slightly widened state and continued to widen with time. The spiracle also widened as the lobes spread (Fig. 4a). The spiracle was observed as a narrow slit at *t* = 26 ms. However, its orifice distinctly opened at 104 ms.

The average distance between the three pointed ends of the lobes (*pl*1, end of the left posterior lobe; *pl*2, end of the right posterior lobe; and *al*, end of the anterior lobe) was obtained (Fig. 4b). The average distance increased during the floating process. The distance mainly increased during the first half period (0–52 ms). The increase rate was lower in the second half.

**Figure 5:**
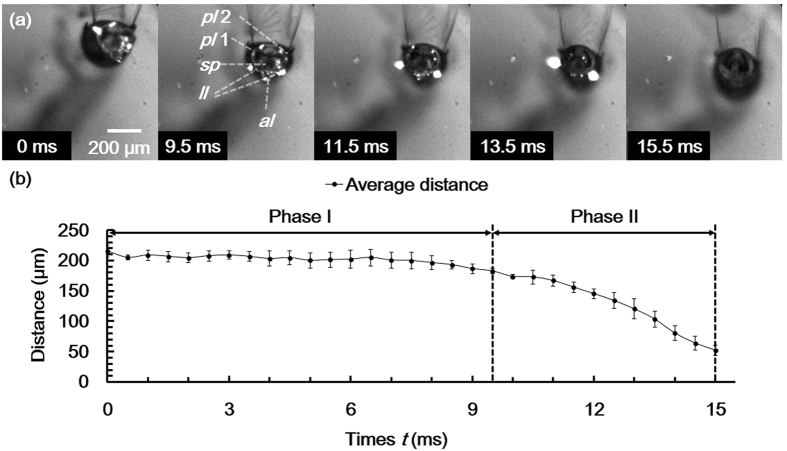
Submerging process of the siphon observed by a high-speed camera. The start (*t* = 0 ms) and end (*t* = 15.5 ms) points are defined as the instants at which the floating siphon starts to move and leaves the water surface, respectively. (**a**) High-speed images extracted at representative instants. (**b**) Temporal variation of average distance (mean ± s. d.) between the tips of the three lobes (*pl*1*, pl*2*, al*) during the submerging process. The submerging process can be divided into two phases. No remarkable contraction of the lobes occurred in phase I. By contrast, the lobes folded in phase II.

The dynamic behavior of the lobes was investigated during submerging process (defined in Methods). It could be divided into two phases as follows. In phase I, the average distance between the tips of the three lobes was not changed noticeably, although the overall position of the apical part of the siphon shifted. Thereafter, the distance noticeably decreased with negligible horizontal shifting in phase II. The lobes lost contact with the water surface at *t* = 15.5 ms (Fig. 5a). The lobes were closed and fully submerged under water. The distance was measured just before the closure of lobes.

**Figure 6:**
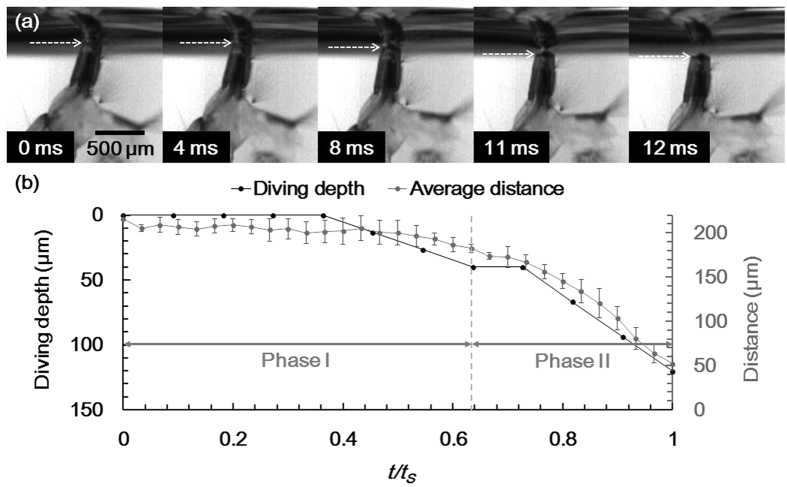
Lateral view of the submerging process of the siphon. Larva leaves the water surface at *t* = 12 ms. (**a**) High-speed images at typical instants. The circular edge of the siphon was pointed by white arrow. The lobes sustain the contact with the water surface up to *t* = 11 ms. (**b**) Diving depth of the circular edge of the siphon was compared with the average distance between the ends of the lobes, in which *t/t*_*S*_ is dimensionless time.

The submerging process was also observed laterally. The circular edge of the siphon end was continuously tracked to measure the diving depth of the siphon. The diving depth of the siphon did not change, but its horizontal position slightly changed during the first 4 ms. Thereafter, the diving depth increased gradually. The increase in depth accelerated at *t* = 8 ms. The lobes were still in contact with the water surface at *t* = 11 ms and then perfectly submerged at *t* = 12 ms (Fig. 6a). The diving depth at *t* = 12 ms was about 147 μm.

To compare with the result observed from the apical view, the average distance between the ends of the three lobes and the diving depth of the siphon edge were depicted according to the dimensionless time, *t/t*_*S*_ (Fig. 6b). The diving depth was plotted while the lobes maintained contact with the water surface. The two variation curves exhibited similar tendencies. Both diving depth and distance were not changed evidently during the first half of phase I. Thereafter, diving depth and distance gradually deepened and decreased, respectively. The rates of change of these parameters were similarly enhanced in phase II.

### Floating mechanism of the siphon

**Figure 7:**
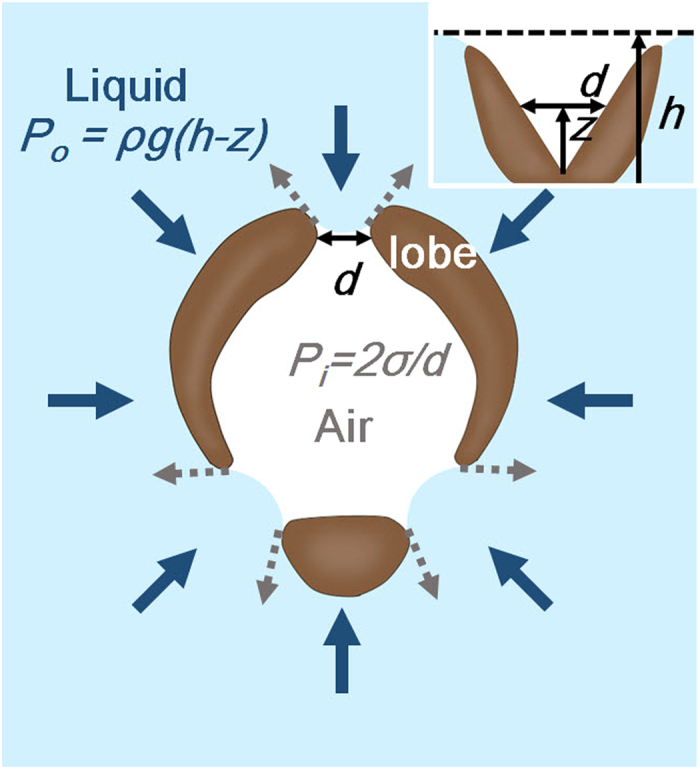
Schematic diagram of partially expanded lobes elucidating the floating and submerging mechanisms. Here, *σ* is the surface tension of water, *ρ* is the density of water, *d* is the gap between neighboring lobes at height *z* from the siphon tube, and *h* is the diving depth of the siphon.

The floating mechanism of the lobes of the mosquito larva was derived based on the morphological structures, wettability, and dynamic behavior of the siphon. Figure 7 shows a cross-section image of the partially expanded lobes with pressures acting on them.

The lobes were compressed by hydrostatic pressure *P*_*o*_* = ρg*(*h*-*z*), depending on the depth *h* of the circular edge of the siphon and its depth-wise coordinate *z* (from the circular edge of the siphon)^16^. In addition, the hydrostatic pressure promoted the intrusion of water through the gaps between lobes. As the diving depth increases, the hydrostatic pressure will increase. Each air-water interface between the lobes would have a negative curvature because of the hydrophobicity of the lobes. Assuming that the curvature had a cylindrical shape, the pressure derived from surface tension can be expressed as *P*_*i*_* = *2*σ*/*d*, where *σ* is the surface tension of water, and *d* is the gap between adjacent lobes^16^. Therefore, the intrusion of water would not occur if *P*_*i*_* ≥ P*_*o*_, and the lobe could be closed safely during the submerging process^16^. If *d* satisfies the following condition:


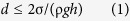


then the condition *P*_*i*_ ≥ *P*_*o*_should be satisfied, because *h* ≥ *h*-*z*. In this study, the lobes were closed at the depth of *h* = 147 μm. The upper limit of *d* for equation (1) was approximately 10 cm, based on the values of *σ* = 0.072 N/m, *ρ* = 1000 kg/m^3^, and *g* = 9.8 m/s^2^. The gap between adjacent lobes should be smaller than 10 cm, because the distance between the pointed ends of the lobes is in the order of 100 μm. Therefore, flooding would not occur during the submerging process.

Similarly, water would not flood through the gap between the lobes during the floating process. The hydrostatic force pressing the lobes was reduced at the points where the lobes touched the water surface. The lobes would be pulled out by the surface tension and hang from the water surface. In this process, the spiracle surrounded by lobes could be safely exposed to air for breathing.

## Discussion

*Aedes* mosquito larvae breathe air by floating their hydrofuge lobes on the water surface, and the surface tension can pull the hydrofuge lobes at the water surface^8^,^11^. In this study, we investigated the floating mechanism of the lobes of mosquito larvae for respiration. The function of the lobes was experimentally examined, especially three main lobes, namely, an unpaired anterior lobe and a pair of posterior lobes. A pair of lateral lobes was smaller than the three main lobes, and they were not clearly observed in X-ray micro-CT images and high-speed optical images. The morphological structures of the three main lobes resembled a hollow cone, which was split into three pieces. The spiracle was placed on the base of the cone (Fig. 1). When the siphon floated on the water surface, the three lobes were spread into a triangular shape in apical view with the spiracle exposed to air. When the siphon was submerged under water, the three lobes contracted into a cone shape to make the spiracle watertight.

The wettability of the lobes is also significant for the floating mosquito larva on the water surface^13^. Typical contact angle of the arthropod cuticle is 105° 6,17,18. Keilin *et al*.^19^ reported that the siphon secretes oil to coat the lobes and make them hydrofuge. In this study, the wettability of the apical parts of siphon was evaluated by observing the air bubble on the lobes. The air bubble released through the spiracle showed a negative curvature like a bulb on the lobes. This phenomenon indicates that the lobes had hydrophobic surface. The lobes tried to hold the air bubble because of their hydrophobicity. The detachment of the air bubble would result in flooding of water. The holding of air bubble led to the bulb shape as the volume of the air bubble increased. The hydrophobicity of the lobes was also tested using Nile red, which is used as fluorescence hydrophobic probe for arthropod mouthpart^14^,^20^. The larva stained with Nile red revealed that the apical part of its siphon has hydrophobic wettability.

The dynamic behavior of the lobes during the floating process was experimentally investigated. The lobes touched the surface of water, and then they continued to widen the gaps between their pointed ends. The hole of the spiracle also increased with the expansion of the lobes. This floating process occurred at the surface of water, and no flooding of water occurred. Thus, the larva was ready to inhale air by opening the lobes at the water surface.

The lobes contracted during the submerging process until they left the water surface. Thus, contraction occurred at the surface of water. When lobes were detached from the water surface, they finished the contraction and sealed the spiracle. In this process, no flooding of water occurred through the gaps between lobes. The temporal variation of the depth of the siphon showed a trend similar to that of the distance between the end points of the lobes.

Therefore, the floating mechanism of the lobes could be explained with the surface tension and hydrostatic pressure caused by geometry, hydrophobicity of lobes, and the depth. The water would make negative curvatures in the gaps between the lobes because of the hydrophobicity of lobes. In addition, the surface tension acting on the gap was sufficient to prevent flooding induced by hydrostatic pressure. Therefore, the lobes would be safely closed while they were pressed by hydrostatic pressure during the submerging process. When the lobes touched the surface of water, they would be pulled out and spread by the surface tension.

The density of a living larva is similar to that of water^8^. The bond number, *Bo = ρ*_*l*_*gl*^2^*/σ* (the ratio of the gravitational force pulling the larva down to the force caused by surface tension holding up) is approximately 5.44 × 10^−3^, where *ρ*_*l*_ is the density of the larva (≈ density of water, 1000 kg/m^3^), and *l* is the character length of the lobes (200 μm). Therefore, the floating of the larva could be sustained when the lobes were spread on the water surface. In addition, the depth where the hydrostatic pressure would be compatible with the pressure induced by the surface tension could be estimated as *H* ~ 2*σ*/*ρgl* based on equation (1). The depth *H* has a value in the order of 10^−2^ m. This value may provide insight into the relationship between mosquito larvae and breeding sites (generally shallow water). The flooding of water might not occur even though the lobes were open within the depth of water ~ 10^−2^ m.

In conclusion, the lobes of the mosquito larva seem to be well adapted to interaction with the water surface because of their opening and closing behavior. The spiracle surrounded by the lobes could be safely exposed to air for breathing and sealed when the lobes arrive and leave the water surface, respectively. This study would be beneficial for elucidating the respiratory behavior of mosquito larvae. Furthermore, the floating mechanism observed in this study could be applied to study other aquatic insects breathing air on the water surface by flapping hairs or valves surrounding spiracles^7^.

## Methods

### Morphological analysis of the lobes

*Aedes togoi* larvae were collected from a shallow pool of rocky seashore in Pohang, South Korea. They were reared in a small water tank located in an air-conditioned room at 27 °C, 80% relative humidity and 16 h/8 h light/dark photoperiod. The fourth instar larvae of *Aedes togoi* mosquito were dehydrated in a series of ethanol solutions (70%, 80%, 90%, and 100%, overnight each), soaked in hexamethyldisilazane liquid and dried in the air. The three dried samples were coated with platinum, and the structures were observed using a field emission scanning electron microscope (XL30 FEG, Philips, the Netherlands).

The three dehydrated specimens were placed on the rotating stage of the 6 C Biomedical Imaging (BMI) beamline of Pohang Accelerator Laboratory (Pohang, Republic of Korea) to obtain 3D tomographic information. X-ray beam passed the siphon of the specimen. X-ray projection images of the siphon were obtained at intervals of 0.5° during rotating stage through 180°. The 3D image of the siphon was analyzed using Amira software (Visualization Science Group, USA) after reconstruction of tomographic image with Octopus software (in CT, Belgium).

The morphology of the siphons of the five live larvae were observed using an optical microscope (Eclipse 80i, Nikon, Tokyo, Japan) with a digital camera (D700, Nikon, Tokyo, Japan).

### Wettability test

The mosquito larva was gently pipetted onto a slide glass with a droplet by using a disposable transfer pipette whose tapered tip was snipped off, and then covered with a cover glass. The air bubble was released from the spiracle of the siphon when its body was slightly compressed by pressing the upper cover glass. The shapes of the air bubbles on the apical part of the siphons of three larvae were observed using a microscope (Eclipse 80i, Nikon, Japan). The wettability of the apical parts of the siphons of three larvae was also investigated using Nile red, a fluorescent hydrophobic probe^14^,^15^. Nile red (Sigma-Aldrich, Germany) was prepared as a stock solution of 500 μg/ml in acetone. The staining solution of Nile red was made by adding 10 μl of stock solution to 1 ml of 75% glycerol (final concentration 5 μg/ml)^14^,^15^. The larvae were stained with Nile red staining solution for 30 minutes. The stained sample was illuminated with Nd: YAG continuous laser (λ = 532 nm, SLOC, Shanghai, China). Fluorescent images of the sample were observed by a microscope (Eclipse 80i, Nikon, Japan) attached with an optical long-pass filter (λ > 550 nm).

### *In vivo* high-speed imaging of floating phenomena

The living larvae were put in a microtube containing water. The tube was placed on the stage of a microscope (Eclipse 80i, Nikon, Japan) attached with a high-speed CMOS camera (Photron Ultima APX, Fujimi, Japan). The microscope focused on the water surface. The dynamic behaviors of the siphons of two floating larvae were recorded in top view at 2000 frames per second (fps). The typical floating process was defined to start when the water surface started to be distorted by contact of the lobes (*t* = 0 ms) and to end when the lobes stopped spreading on the water surface (*t* = 105 ms). The two submerging larvae were also filmed in the top view at 2000 fps. The typical submerging process was defined to begin when the larva started from the floating state. The end point of the process was when lobes lost contact with the water surface (Figs 5a and 6a).

In addition, living larvae were put into water in an acrylic cube. The high-speed camera attached with a microscopic lens (Plan 4×, Nikon, Japan) recorded the submerging process of two larvae from the side of the cube at 1000 fps.

To analyze the dynamic behaviors of the lobes, the distances between the lobes and diving depth of the siphon were obtained using manual tracking method with ImageJ. We enhanced image contrast of the captured high-speed images. The end positions of each lobe were manually tracked at pixel resolution. The spatial resolution of pixel was about 4.22 μm. The distance between the two end positions was then calculated. At some instances, the scattered light from the tip region of the lobes hid the end position. In this case, we chose the center of the scattered light. The diving depth of the siphon was also obtained by manually tracking its circular edge. The spatial resolution of pixel was about 13.4 μm.

## Additional Information

**How to cite this article:** Lee, S. C. *et al*. Floating of the lobes of mosquito (*Aedes togoi*) larva for respiration. *Sci. Rep.* **7**, 43050; doi: 10.1038/srep43050 (2017).

**Publisher's note:** Springer Nature remains neutral with regard to jurisdictional claims in published maps and institutional affiliations.
